# Improvement of BCI performance with bimodal SSMVEPs: enhancing response intensity and reducing fatigue

**DOI:** 10.3389/fnins.2025.1506104

**Published:** 2025-03-06

**Authors:** Junjie Liu, Jun Xie, Huanqing Zhang, Hanlin Yang, Yixuan Shao, Yujie Chen

**Affiliations:** ^1^School of Mechanical Engineering, Xinjiang University, Urumqi, China; ^2^School of Mechanical Engineering, Xi'an Jiaotong University, Xi'an, China; ^3^State Key Laboratory for Manufacturing Systems Engineering, Xi'an Jiaotong University, Xi'an, China

**Keywords:** brain-computer interface (BCI), steady-state motion visual evoked potential (SSMVEP), bimodal motion-color stimuli, augmented reality (AR) glasses, signal-to-noise ratio

## Abstract

Steady-state visual evoked potential (SSVEP) is a widely used brain-computer interface (BCI) paradigm, valued for its multi-target capability and limited EEG electrode requirements. Conventional SSVEP methods frequently lead to visual fatigue and decreased recognition accuracy because of the flickering light stimulation. To address these issues, we developed an innovative steady-state motion visual evoked potential (SSMVEP) paradigm that integrated motion and color stimuli, designed specifically for augmented reality (AR) glasses. Our study aimed to enhance SSMVEP response intensity and reduce visual fatigue. Experiments were conducted under controlled laboratory conditions. EEG data were analyzed using the deep learning algorithm of EEGNet and fast Fourier transform (FFT) to calculate the classification accuracy and assess the response intensity. Experimental results showed that the bimodal motion-color integrated paradigm significantly outperformed single-motion SSMVEP and single-color SSVEP paradigms, respectively, achieving the highest accuracy of 83.81% ± 6.52% under the medium brightness (*M*) and area ratio of *C* of 0.6. Enhanced signal-to-noise ratio (SNR) and reduced visual fatigue were also observed, as confirmed by objective measures and subjective reports. The findings verified the bimodal paradigm as a novel application in SSVEP-based BCIs, enhancing both brain response intensity and user comfort.

## 1 Introduction

A brain-computer interface (BCI) system translates brain activity patterns into commands for interactive applications (Anumanchipalli et al., 2019; Heelan et al., 2019; Lamti et al., 2019; Spiegel et al., 2019; Kubanek et al., 2020). The steady-state visual evoked potential (SSVEP) paradigm is a widely used BCI stimulation method, featured by its ability of providing multiple commands, requiring few EEG electrodes, and offering robust anti-interference properties. Compared to other BCI methods such as motor imagery (MI) (Li et al., 2023; Phang and Ko, 2020; Khademi et al., 2023), P300 event-related potential (ERP) (Yin et al., 2015a), and transient visual evoked potential (tVEP), SSVEP BCI does not necessitate extensive training, and can achieve high recognition accuracy (Yin et al., 2015b). However, traditional SSVEP paradigms, which typically employ light flicker or graphic flipping for stimulation, often lead to visual fatigue and discomfort, resulting in an underlying decrease in recognition accuracy.

Recently, to prevent the adverse effects of sustained intense stimulation on brain neurons, researchers have developed motion-based BCI paradigms (Snowden and Freeman, 2004). Motion-elicited visual evoked potentials (mVEPs) are divided into transient and steady-state categories (Vialatte et al., 2010). Guo et al. (2008) and Hong et al. (2009) presented transient N2 based BCI systems relying on motion detection. These paradigms showed notable benefits in VEP-based BCI research by utilizing consistent luminance and non-flickering methods. However, it also has limitations as the transient approach requires multiple stimulation targets to move in a single direction, which may result in motion after-effect (MAF) (Hammond et al., 1986).

To overcome these challenges, Xie et al. (2012, 2014) designed a BCI paradigm based on steady-state motion visual evoked potential (SSMVEP) utilizing Newton's rings, which enhanced recognition accuracy. However, maintaining uniform brightness in the central region of Newton's rings during motion is still difficult, resulting in low signal-to-noise ratios (SNR) in frequency peaks.

SSVEP primarily depends on the human visual system's (HVS) sensitivity to variations in light intensity. The HVS, consisting of the retina, lateral geniculate nucleus (LGN), and visual cortex, is a sophisticated system. The visual cortex, encompassing the primary visual cortex (V1) and extrastriate regions (e.g., V2, V3, V4, V5/MT), is part of the advanced central neural network (Lu et al., 2023; Kam and Chang, 2023). The dorsal stream, or the M-pathway, is essential for motion detection and spatial analysis, with identifying the velocity and direction of movement (Gmel et al., 2023). The ventral stream, also known as the P-pathway, is responsible for color vision and object identification, with discerning luminance and color (Kravitz et al., 2013). When visual stimuli have two colors with equal brightness, flicker sensitivity in the eyes is diminished (Nagai et al., 2022).

In tasks where specific targets need to be found, the ability to quickly identify targets is influenced by various factors, one of which is the understanding of the target's specific characteristics. This helps more effectively to catch attention, and more likely to focus on the location of the target. These key factors include certain static or dynamic features, such as color changes, the appearance of targets, etc. When there is a target or event that cannot be ignored by the brain, this phenomenon is considered as “attentional capture” (von Mühlenen and Conci, 2016). As long as they are unique in time, any local changes can attract attention. Therefore, local color changes can make it easier for subjects to increase attention, so color changes can be added to the SSMVEP paradigm to achieve the effect of enhancing attention.

To overcome the limitations of traditional SSVEP paradigms, we propose an innovative SSMVEP framework that integrates color contrast into the Newton's rings design. This framework combines motion patterns with color variations to boost SSMVEP responses while minimizing discomfort. By analyzing the principles of AR presentation, we designed experiments to refine the paradigm parameters and examined the effects of color contrast on multi-ring SSMVEP paradigms at low frequencies. This method aims to provide a new solution for improving SSMVEP paradigms in AR environments.

We hypothesize that equal-luminance color contrast in Newton's rings based visual stimulation can activate more neuronal responses in the visual cortex of M- and P-pathways, resulting in higher SNRs of SSMVEP responses. This study developed a color-contrast Newton's rings paradigm that incorporates color, shape, luminance, and motion characteristics. The aim was to determine if this equal-luminance color-contrast paradigm could evoke distinct SSMVEPs with improved SNR, thereby enhancing BCI interactive performance.

## 2 Materials and methods

### 2.1 Subjects

Ten subjects (six males and four females) from Xi'an Jiaotong University, with an average age of 25 years (±3 years), were recruited for the study. All had either normal or corrected-to-normal vision and hearing and were experienced with SSVEP-BCIs before. None had a history of visual or auditory disorders, and none received compensation for their participation. The study adhered to the principles of the Declaration of Helsinki. Each subject gave written informed consent, following the protocols sanctioned by the institutional review board of Xi'an Jiaotong University.

### 2.2 EEG recordings

Using the International 10–20 electrode placement system (Homan et al., 1987), we recorded six-channel EEG recordings from the parietal and occipital regions, specifically at locations Po3, Poz, Po4, O1, Oz, and O2. The g.USBamp device (g.tec, Graz, Austria) was used for the recordings at a 1,200 Hz sampling rate. To minimize environmental background noise, subjects were asked to sit in front of a vertically placed black cloth during the experiments. EEG recordings were referenced to one earlobe and the ground electrode was at the Fpz location, with resistance levels maintained under 5 kOhm. The EEG responses first underwent analog filtering, followed by digital filtering with an 8th-order Butterworth band-pass filter to retain frequencies from 2 to 100 Hz. A 4th-order Butterworth notch filter was also used to eliminate powerline interference in the 48–52 Hz range.

### 2.3 Stimulation design

The multi-ring SSMVEP paradigm consists of rings with low contrast brightness against a high contrast background. The main parameters of the rings include inner and outer diameters. The high contrast background determines the maximal diameter of the rings. The area ratio between the rings and the background is defined as the following relationship:


(1) $$\begin{array}{l} C=\frac{S_{1}}{{S-S}_{1}} \end{array}$$

where, *S*_1_ is the total area of the rings, and *S* is the total area of the background.

Initially, the outer diameters of the rings are set in an arithmetic sequence. Given the maximum diameter *r*_max_, the relationship between the outer diameter *r*_i_ and the maximum diameter *r*_max_ of each ring can be defined as:


(2) $$\begin{array}{l} r_{i}=\left(2i-1\right)\cdot \frac{r_{\max}}{2n} \end{array}$$

where, *i* is the index of the ring and *n* is the total number of rings.

The inner radius *r*_*i*−1_ can be calculated using the area ratio *C* and the following relationship:


(3) $$\begin{array}{l} r_{i-1}^{2}\;=\;\left(1-C\right)\cdot r_{i}^{2}\;+\;C\cdot r_{i-1}^{2} \end{array}$$

The multi-ring paradigm elicits SSMVEPs through the movement of rings. As the rings move, their colors and the background color change simultaneously, presenting a color modulation mode. In this study, red and green were chosen as the alternating colors. To avoid flickering, the color was designed to be gradually and smoothly varied. Thus, black color would change to red color and white color would change to green color (Anumanchipalli et al., 2019).

The changes of color affect the brightness, which could enhance the paradigm's stimulation effect. Therefore, it is crucial to maintain constant luminance across the visual fields of the subjects. The following equation is used to calculate perceived luminance:


(4) $$\begin{array}{l} L\left(r,g,b\right)\;=\;C_{1}\left(0.2126R+0.7152G+0.0722B\right) \end{array}$$

where, *L*(*r, g, b*) represents perceived luminance, *C*_1_ is a constant value determined by the presentation device used. For this study, *C*_1_ was selected as 0.7, which provides a good balance between contrast and smoothness for the specific characteristics of the AR glasses utilized, and *R, G*, and *B* denote the red, green, and blue color values.

As the stimulation starts, the color of the rings begins to change. To ensure smooth contrast change, a sine wave function is employed. The color value of the rings at any given time is calculated as follows:


(5) $$\begin{array}{l} R\left(t\right)\;=\;R_{\max}\left(1-cos\left(2\pi ft\right)\right) \end{array}$$

where, *R*_max_ is the maximum brightness, *f* is the frequency, and *t* is the time.

**Figure 1:**
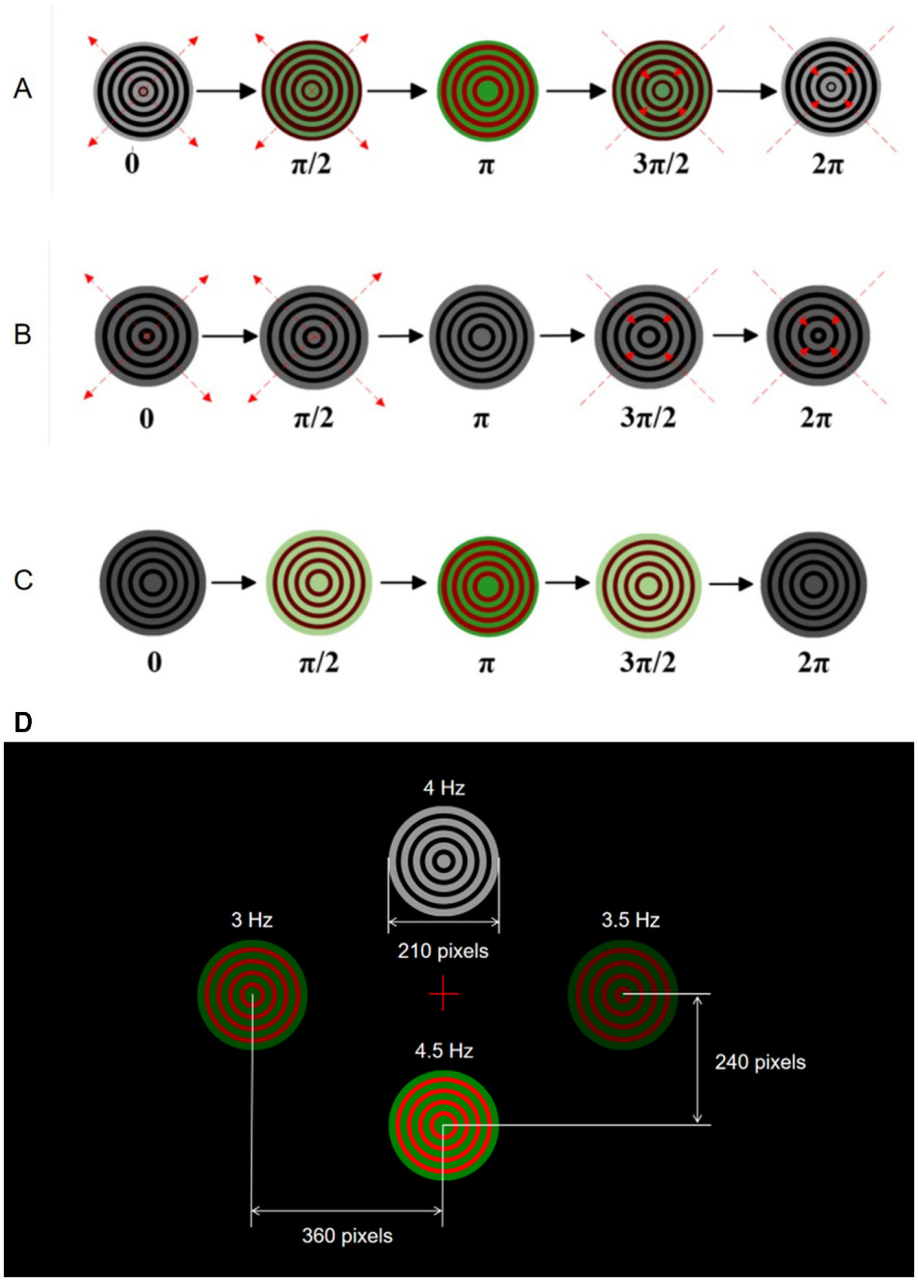
Visual stimulation paradigms for SSMVEP experiments. **(A)** Bimodal Newton's rings combining color and motion. **(B)** Monochrome motion Newton's rings. **(C)** Color change Newton's rings without motion. **(D)** AR interface displaying four stimulus points at different frequencies.

Figure 1 illustrates the movement patterns within one motion cycle for three different Newton's rings paradigms used in the experiments. Figure 1A represents the bimodal Newton's rings paradigm combining motion and color, where the rings alternately change colors between red and green while expanding outward from 0 to π and contracting inward from π to 2π. Figure 1B shows the monochrome motion paradigm, featuring black and white Newton's rings that expand outward from 0 to π and contract inward from π to 2π without any color change. Figure 1C depicts the paradigm with only color changes, where the rings alternate between red and green without any motion. Each paradigm demonstrates complete motion cycles to present the stimulation to subjects, providing a comparison between the three approaches.

The AR interface in the experiments displayed four targets through AR glasses, designed to elicit EEG responses at 3, 3.5, 4, and 4.5 Hz. Subjects wore AR glasses to experience consistent visual stimuli. Due to the low refresh rate of AR glasses, the low stimulation frequency band was chosen in this study. In addition, the low stimulation frequency band can reduce visual discomfort and corresponding fatigue, while maximizing the eliciting effect of EEG responses under the current AR glasses device (Vialatte et al., 2010).

In the AR interface, the four targets are arranged in a specific layout as the top (i.e., 4 Hz) and bottom (i.e., 4.5 Hz) targets are positioned vertically 240 pixels from the center of the screen, and the left (i.e., 3 Hz) and right (i.e., 3.5 Hz) targets are 360 pixels from the center of the screen horizontally. As shown in Figure 1D, this arrangement ensures clear distinguishability and an unobstructed field of view for subjects. The visual stimuli are represented as concentric rings that either change colors or expand and contract according to their specific paradigms. This setup ensures that subjects receive consistent and controlled visual stimuli throughout the experiments, allowing for accurate measurement of EEG responses.

**Figure 2:**
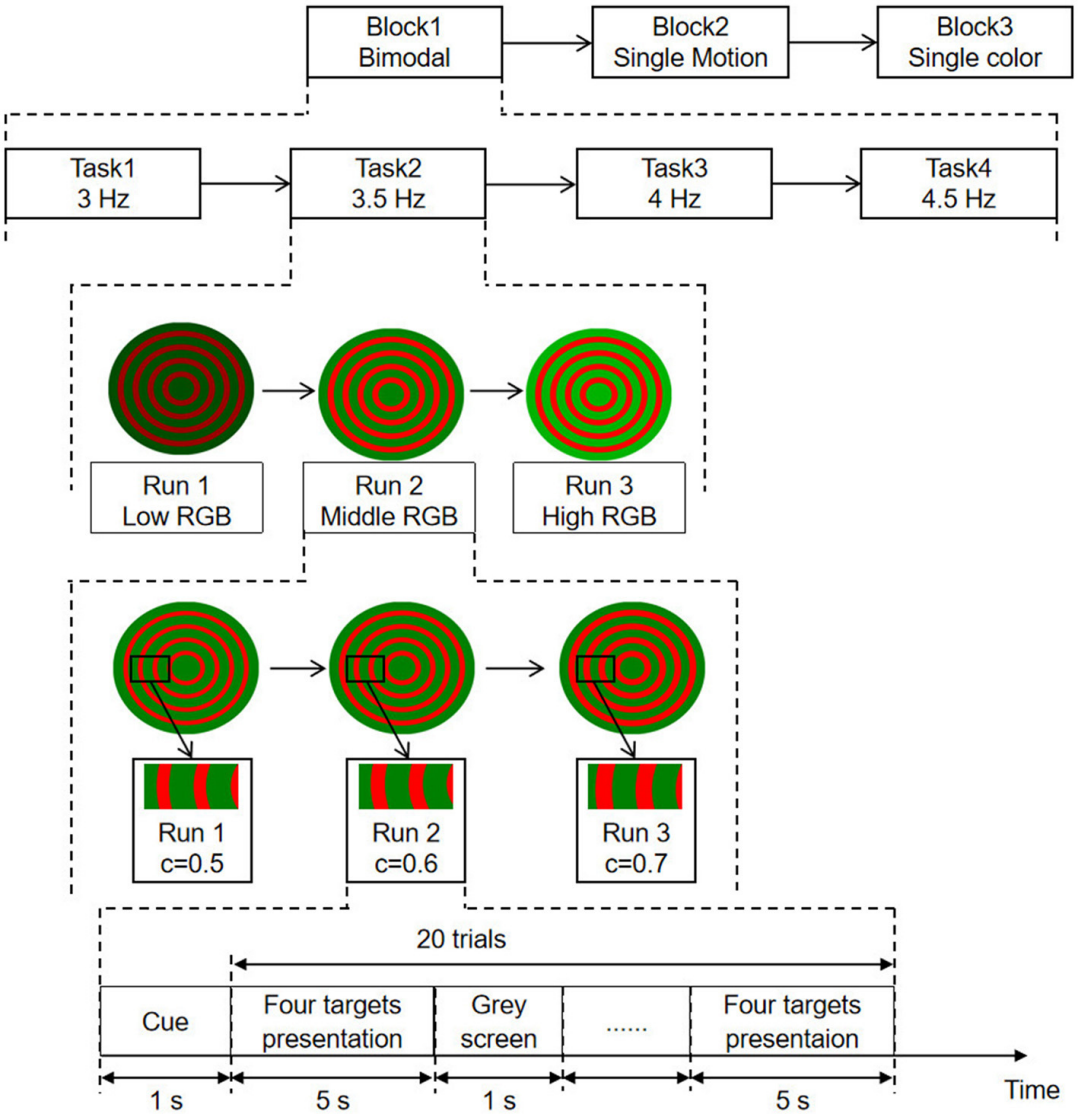
Experimental procedure for SSMVEP paradigms. Sessions include bimodal, single-motion, and single-color paradigms. Tasks involve frequencies of 3, 3.5, 4, and 4.5 Hz. Runs vary in brightness (low, middle, high RGB) and contrast levels (*C* = 0.5, 0.6, 0.7). Each trial consists of cue, target presentation, and gray screen intervals.

**Figure 3:**
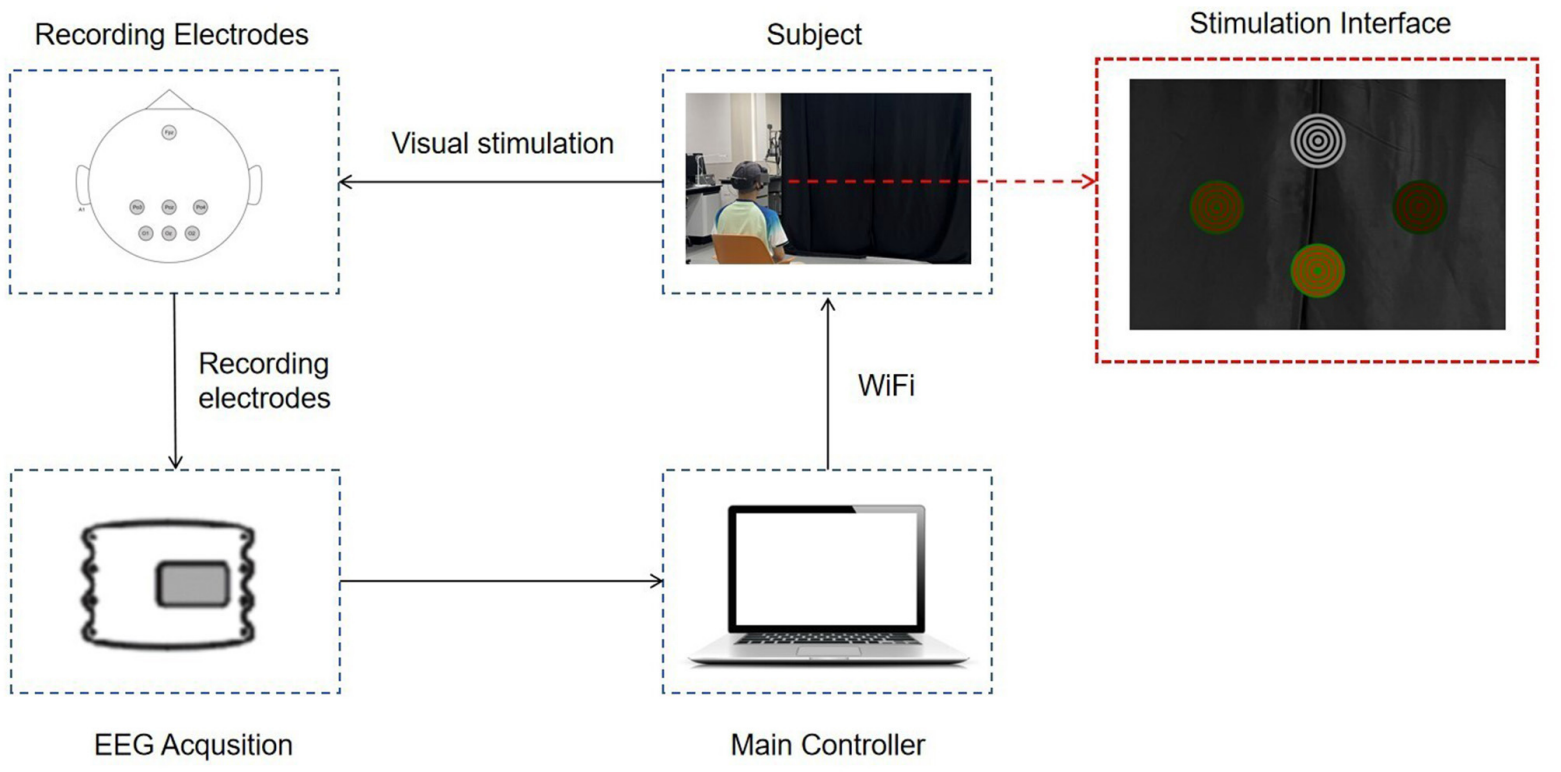
The overall BCI system diagram. The subject wears AR glasses displaying the stimulation interface. Visual stimulation is presented, and EEG signals are recorded using electrodes. Data is transmitted via WiFi to the main controller for processing and analysis.

The experimental procedure, shown in Figure 2, comprises three blocks as bimodal SSMVEP, single-motion SSMVEP, and single-color SSVEP paradigms. Each block includes four tasks with respect to stimulation frequencies of 3, 3.5, 4, and 4.5 Hz, corresponding to task 1, task 2, task 3, and task 4, respectively. Task 1 to task 4 are carried out sequentially. Each task is further divided into three runs, and each run representing different brightness levels (i.e., low, medium, and high) and varying contrast levels (i.e., *C* = 0.5, *C* = 0.6, and *C* = 0.7, where *C* represents the area ratio). During each run, subjects were presented with four targets simultaneously and were instructed to focus on one specific target designated by the operator. Each trial lasts for 5 s, with a 1-s cue and a 1-s gray screen as the inter-trial interval. To ensure the subjects' comfort and the reliability of the experimental results, a rest period of at least 2 min is provided after completing each block. During this time, subjects can adjust their posture, blink, and perform other necessary actions. After resting, subjects will restart the experiments by clicking the start button. The complete BCI configuration is shown in Figure 3.

### 2.4 Data preprocessing

The acquired responses from each subject were preprocessed offline. EEG segments corresponding to each run were extracted, resulting in a data matrix of 10 subjects × 3 brightness levels × 4 stimulation frequencies × 20 trials × 6 channels × 6,000 sampling points. The responses from all trials were then combined and averaged, followed by band-pass filtering of 1–30 Hz to remove low-frequency artifacts and high-frequency interferences. Subsequently, the data were examined using Welch's power spectral density (PSD) method and EEGNet as described below.

### 2.5 SNR analyses

PSD is a commonly used method in signal processing to describe the power distribution of a signal across different frequency range (Zhang et al., 2023; Kang et al., 2020). It represents the energy distribution of a signal in the frequency domain and can be used to analyze the spectral characteristics of the signal. The definition of PSD is as follows:


(6) $$\begin{array}{l} S_{x}\left(k\right)={\frac{2\pi }{N}|\overset{N-1}{\underset{n=0}{\sum }}X\left(n\right)e^{-i\left(2\pi /N\right)kn}|}^{2}\;,0\leq k\leq N-1 \end{array}$$

where, *N* is the number of sampling points.

The SNR is a measure of the relative strength of the signal to noise and is used to describe the intensity of the signal (Kim et al., 2023). It represents the ratio of signal power to noise power and is expressed using the PSD. The equation for SNR is as follows:


(7) $$\begin{array}{l} SNR=10log_{10}\frac{\overset{n}{\underset{l=1}{\sum }}S_{x}\left(\frac{lfN}{F_{s}}\right)}{\overset{N}{\underset{j=0}{\sum }}S_{x}\left(k\right)-\overset{n}{\underset{l=1}{\sum }}S_{x}\left(\frac{lfN}{F_{s}}\right)} \end{array}$$

where, *n* is the number of harmonics, *F*_*s*_ is the sampling frequency, *f* is the stimulation frequency.

### 2.6 EEGNet method

**Figure 4:**
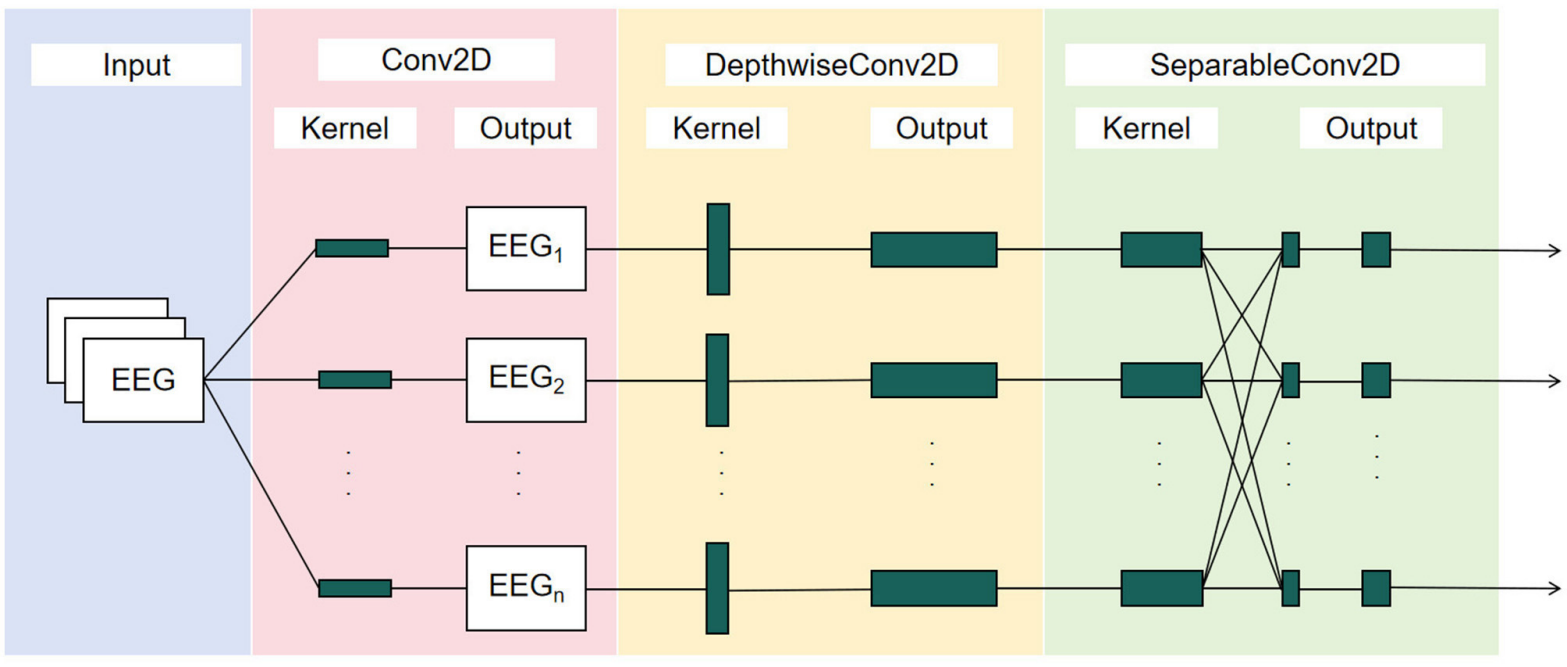
EEGNet architecture.

EEGNet is a compact convolutional neural network architecture specifically designed for EEG signal processing and brain-computer interface BCI systems. Its streamlined structure and high efficiency enable it to adapt to a wide range of BCI tasks (Lawhern et al., 2018), is illustrated in Figure 4. Therefore, we selected this model as the SSVEP classification tool for the present study. It consists of three essential network components as conventional convolutional layers, depth-wise convolutional layers, and separable convolutions. These elements extract and refine the critical features necessary for complex neural response classification.

The input layer of the neural network receives EEG data matrix. Initially, this data is processed by a two-dimensional convolutional layer. The kernel size of this layer is determined by the sampling rate of the EEG responses, with L set to half the sampling frequency (i.e., 600), resulting in a convolution kernel dimension of *L*×*1*. This design enables the row-wise filtering of EEG responses, performing channel-wise filtering and generating feature maps through an activation function. This process is mathematically expressed as:


(8) $$\begin{array}{l} y_{j}=f\left(\left(X\;*\;w_{j}\right)+b_{j}\right) \end{array}$$

where, *y*_*j*_ is the *j*-th feature map, *X* is the input signal, *w*_*j*_ is the weight matrix of the *j*-th convolutional kernel, *b*_*j*_ is the bias value, and *f* is the activation function.

Following the initial convolutional layer, the network includes a depthwise convolutional layer. Here, the convolutional kernel's dimensions are equal to the number of channels. Unlike the initial layer, each kernel in the depthwise convolutional layer corresponds to a single input feature map, maintaining a depth of 1. This can be mathematically described as:


(9) $$\begin{array}{l} y_{j}=f\left(\underset{h}{\sum }\left(X_{j,h}\cdot w_{j,h}\right)+b_{j}\right) \end{array}$$

In this equation, *y*_*j*_ represents the *j*-th feature map, *X* is the input signal, *w*_*j*_ is the weight matrix of the *j*-th convolutional kernel, *b*_*j*_ is the bias term.

The separable convolutional layers perform spatial and temporal filtering. By separating these stages, EEGNet efficiently processes EEG responses, capturing intricate patterns within the neural data to ensure robust classification of EEG responses.

In general, the initial convolutional layer performs channel-wise filtering to produce feature maps. These feature maps are further processed by depthwise convolutional layers, which enhance the SNR by focusing on specific frequency components. Finally, separable convolutional layers refine the features by separating spatial and temporal filters, ensuring robust classification of EEG responses.

### 2.7 Statistical analyses

The classification accuracies for each subject across different paradigms (i.e., bimodal, single-color, and single-motion) under different brightness levels and contrast levels were analyzed using one-way ANOVA (Gelman, 2005). Statistical significance was determined at *p* < 0.05 with Bonferroni adjustment applied for multiple comparisons. Results were presented as mean ± standard deviation (SD).

## 3 Results

### 3.1 The influence of paradigm parameters for recognition accuracies

**Figure 5:**
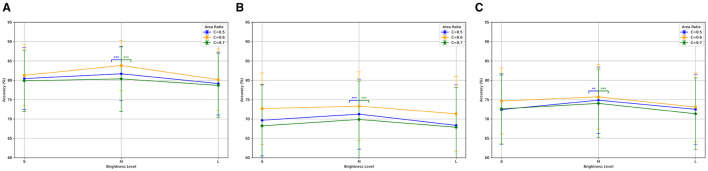
Classification accuracy for three SSMVEP paradigms under different brightness levels and area ratios. **(A)** Bimodal: highest accuracy at medium brightness (M) and *C* = 0.6. **(B)** Single-color: best accuracy at medium brightness (M) and *C* = 0.6. **(C)** Single-motion: highest accuracy at medium brightness (M) and *C* = 0.6. **p < 0.01 indicates a significant difference, and ***p < 0.001 indicates a highly significant difference.

Figure 5A shows the classification accuracies of the bimodal paradigm under different brightness levels and area ratio configurations. The results indicate superior classification performance across all configurations, especially at medium brightness (*M*) and an area ratio of *C* = 0.6 with an average accuracy of 83.81% ± 6.52%. Significance analysis revealed that the accuracy differences between *C* = 0.6 and both *C* = 0.5 and *C* = 0.7 at medium brightness (*M*) were statistically significant (*p* < 0.001). Figure 5B presents the classification accuracies of the single-color paradigm. Despite overall lower performance compared to the bimodal paradigm, the single-color paradigm showed the average accuracy of 73.31% ± 8.88% under the same conditions (*p* < 0.01). Figure 5C displays the classification accuracies of the single-motion paradigm, which outperformed the single-color paradigm but were still inferior to the bimodal paradigm, with an average accuracy of 75.72% ± 8.34% under the same conditions (*p* < 0.01).

The results demonstrated the advantage of the bimodal paradigm in eliciting SSMVEP responses, particularly under specific brightness and area ratio configurations. In the subsequent experimental analysis, we will focus on the data obtained at medium brightness (*M*) and an area ratio of *C* of 0.6 to further explore its potential in maximizing SSMVEP classification accuracy.

### 3.2 Response amplitudes evaluation

**Figure 6:**
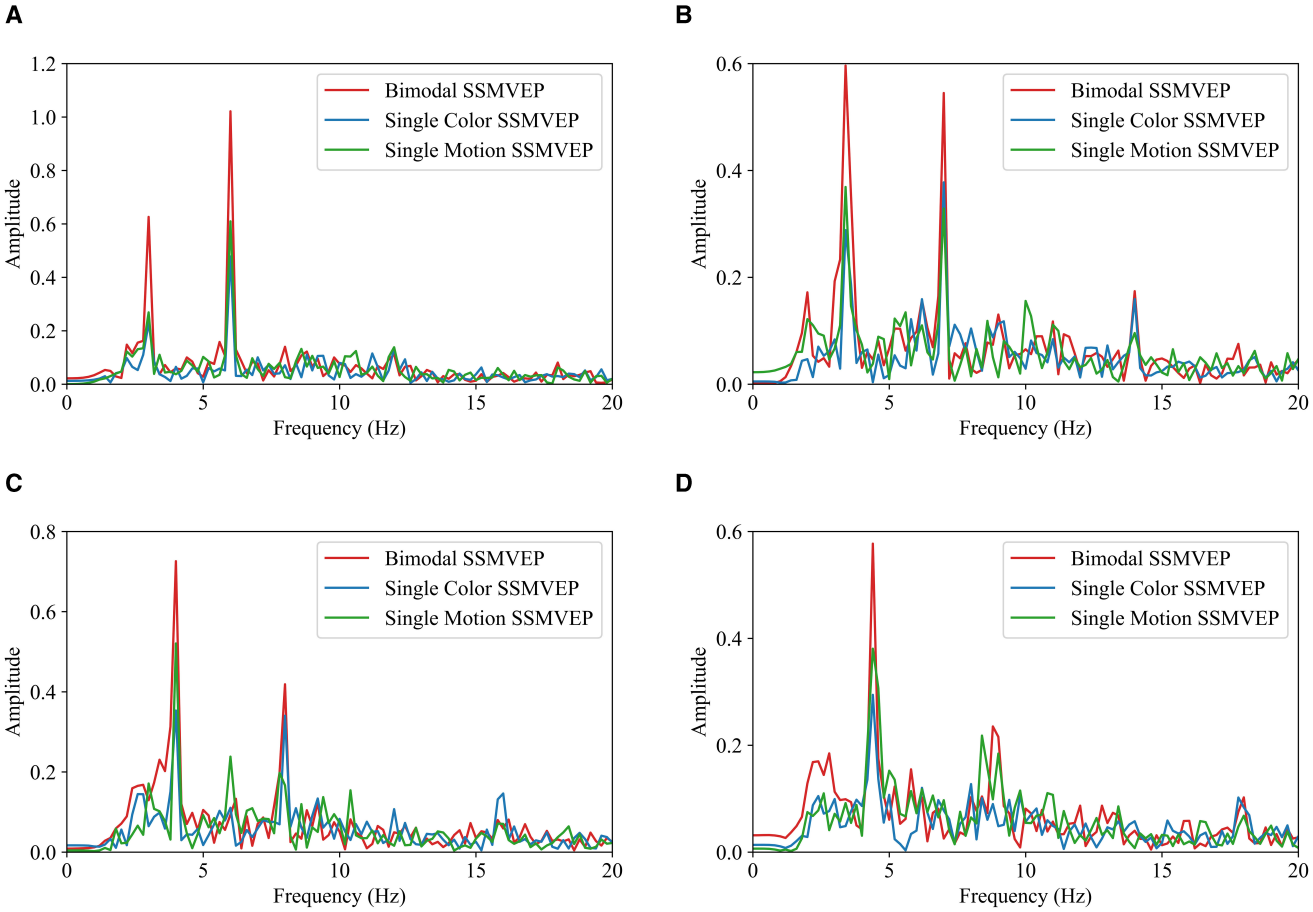
FFT comparison of SSMVEP paradigms under different frequencies. **(A)** 3 Hz. **(B)** 3.5 Hz. **(C)** 4 Hz. **(D)** 4.5 Hz.

EEG responses from the bimodal paradigm with the medium brightness (*M*) and an area ratio of 0.6 were further analyzed to compare with the responses from other paradigms. The fast Fourier transform (FFT) analyses (Ravi et al., 2019) were used for evaluating the frequency characteristics of EEG responses. It helps identifying dominant frequency components and harmonics. Figure 6 presents FFT results at the four stimulation frequencies from all 10 subjects.

EEG data from 10 subjects were filtered using a 4th-order Butterworth band-pass filter with a frequency range of 2–40 Hz. The filtered data were averaged, and FFT was performed to obtain the corresponding frequency spectrum. Across all four frequencies, the SSMVEPs from the bimodal paradigm consistently show the most prominent peaks at the fundamental frequencies, indicating robust evoking effect. This suggests that the bimodal paradigm is more effective in eliciting EEG responses compared to the single-motion and single-color paradigms. Notably, the SSMVEPs from the bimodal paradigm also display obvious second harmonic components, further emphasizing its reliability. Generally, the single-motion paradigm outperformed the single-color paradigm, with discernible peaks at the fundamental frequencies, although these peaks are not as pronounced as those in the bimodal paradigm. The single-color paradigm exhibits the lowest performance, with minimal SSMVEP amplitudes at both the fundamental and harmonic frequency components. Overall, the FFT analyses consistently demonstrates the efficacy of the bimodal SSMVEP paradigm in eliciting steady-state EEG responses. By combining motion and color changes, the bimodal paradigm leverages their synergistic effects to enhance SSMVEP responses, indicating its robustness and reliability for the application of SSMVEP-based BCIs.

### 3.3 PSD based SNRs evaluation

**Figure 7:**
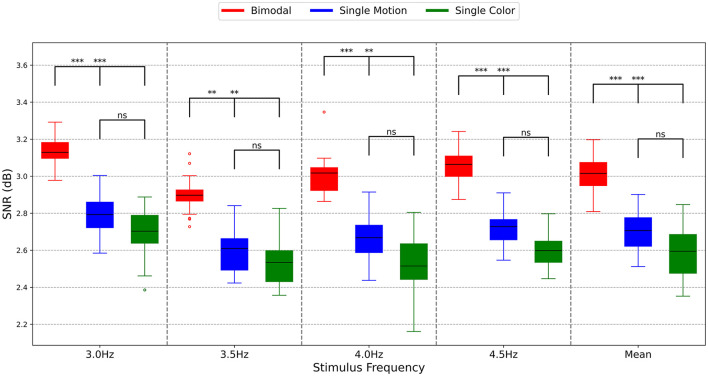
SNRs of different paradigms. The symbols represent the statistical significance levels derived from the Mann-Whitney U test, as follows: ^**^p < 0.01 (highly significant), ^***^p < 0.001 (extremely significant), ns: Not significant (*p* ≥ 0.05). These annotations indicate the significance of differences between the SNR values of the Bimodal, Single Motion, and Single Color methods at each stimulus frequency.

To determine the response intensity, the SNR was calculated using PSD with the number of harmonics set to 4. The average SNRs for the 10 subjects are illustrated in Figure 7. In Figure 7, the red color represents the bimodal paradigm, the blue color represents the single-color paradigm, and the yellow color represents the single-motion paradigm.

As shown in Figure 7, the interquartile range lines for the SNRs of the bimodal paradigm are consistently higher than those of the single-motion and single-color paradigms across all four stimulation frequencies and the mean results. This indicates that the majority of SNR values in the bimodal paradigm surpass those of the other two paradigms, highlighting its greater effectiveness. Particularly at the stimulation frequencies of 3.0 and 4.5 Hz, the SNRs of the bimodal paradigm were distinctly higher than those of the single-motion and single-color paradigms (*p* < 0.001). At the stimulation frequencies of 3.5 and 4.0 Hz, the SNRs of the bimodal paradigm were significantly higher than those of the single-motion and single-color paradigms (*p* < 0.01). This is consistent with the response amplitude results in this study, reinforcing the robustness of the bimodal paradigm. Conversely, across all four stimulation frequencies and the mean results, there was no significant difference in SNR values between the single-motion and single-color paradigms (*p* > 0.05). This analysis underscores that the bimodal paradigm not only enhances the corresponding response amplitudes but also achieves higher signal quality, making it a more reliable and effective approach compared to the single-motion and single-color paradigms.

### 3.4 Cognitive load and fatigue performance

When cognitive load increases, the power of θ rhythm in EEG increases, while the power of α rhythm decreases. An increase in fatigue leads to a reduction in β rhythm and an enhancement in α and θ rhythms. Here, the θ*/*α ratio was used to measure changes in cognitive load, and the (θ+α)*/*β ratio was employed to evaluate the paradigm-related fatigue (Diez et al., 2024; Azadi Moghadam and Maleki, 2023). The calculated ratio values for cognitive load and fatigue were normalized by subtracting the mean and dividing by the standard deviation for each subject to reduce variability due to differences in EEG amplitudes among subjects and to ensure comparability across different individuals.

As described before, three blocks of experiments corresponded to bimodal SSMVEP, single-motion SSMVEP, and single-color SSVEP paradigms, respectively. Each block includes four tasks with respect to stimulation frequencies of 3, 3.5, 4, and 4.5 Hz, corresponding to task 1, task 2, task 3, and task 4, respectively. Task 1 to task 4 are carried out sequentially. The ratio values were linearly fitted from task 1 to task 4 to characterize the evolution process of cognitive load and fatigue over time. The relative difference between the linearly fitted ratio values of task 4 and task 1 was calculated as the variation extents (%) of the cognitive load and fatigue over time, as presented in Table 1.

**Table 1:** The variation extents (%) of the cognitive load and fatigue of three different paradigms.

**Subject**	**Bimodal**		**Single color**		**Single motion**	
	**Cognitive load**	**Fatigue**	**Cognitive load**	**Fatigue**	**Cognitive load**	**Fatigue**
S1	19.4	33.3	35.8	23.4	9.7	3.8
S2	26.6	11.8	41.4	12.3	31.0	5.4
S3	23.8	9.6	17.3	10.2	11.0	39.4
S4	35.9	31.7	−8.1	0.4	−16.8	14.5
S5	13.5	6.4	−38.0	28.7	14.9	−6.7
S6	−49.5	−33.6	47.1	17.2	23.2	2.3
S7	−55.2	−43.1	−48.6	−46.4	−1.2	−25.5
S8	−45.4	500.3	−37.0	125.9	4.0	180.4
S9	−45.7	−40.3	−44.9	−2.0	−25.7	−5.9
S10	−16.4	−0.3	22.4	9.2	38.0	−13.7
Mean	−7.4	7.3	−5.1	8.2	5.9	9.9

As in Table 1, for the bimodal and single-color paradigms that both contain color change modalities, the θ*/*α ratios, which represent the extents of the cognitive load, decreased by 7.4 and 5.1% in average from task 1 to task 4, respectively. Conversely, the cognitive load of the single-motion paradigm increased by 5.9% in average from task 1 to task 4. This demonstrates the effectiveness of the color modality in reducing the cognitive load. Particularly, the cognitive load of Subjects S6 and S7 decreased by 49.5% and 55.2% from task 1 to task 4, respectively, under the bimodal paradigm. While Subject S5's cognitive load decreased by 38.0% under the single-color paradigm, indicating a significant reduction in cognitive load when color modalities were present. In addition, Subject S2's cognitive load increased by 31.0% under the single-motion paradigm, suggesting that cognitive load may increase without color changes.

In terms of fatigue, the average increase from task 1 to task 4 was 7.3% under the bimodal paradigm, which is lower than the average increases of 8.2 and 9.9% observed in the single-color and single-motion paradigms, respectively. Exceptionally, Subject S8's fatigue increased by 500.3% under the bimodal paradigm, compared to an increase of 125.9% under the single-color paradigm and 180.4% under the single-motion paradigm, respectively. While this individual case demonstrates an extreme change in fatigue, the majority of subjects displayed relatively consistent fatigue changes.

To comprehensively evaluate the effects of different paradigms on cognitive load and fatigue, both Mixed-Effects Models and Analysis of Variance (ANOVA) were employed. The Mixed-Effects Models accounted for both fixed effects (paradigm types) and random effects (individual differences among subjects), providing a robust analysis of the data. Following the Mixed-Effects Models, a one-way ANOVA was conducted to further assess the significance of paradigm effects on cognitive load and fatigue, offering *F*-values, degrees of freedom (df) and partial eta squared (η^2^) to quantify the effect sizes.

The findings from the mixed-effects models and the analyses of variance (ANOVAs) are summarized in Table 2. With regard to cognitive load, the mixed-effects model indicated that, compared to the dual-modal paradigm, the single-color paradigm produced a significantly smaller change in cognitive load [*b* = +2.30, 95% CI (+0.514, +3.161), *p* = 0.029], whereas the single-motion paradigm resulted in a significantly greater increase in cognitive load [*b* = +13.30, 95% CI (+10.308, +16.354), *p* = 0.016]. An ANOVA confirmed the significant effect of paradigm on cognitive load, *F*_(2, 18)_ = 15.67, η^2^ = 0.64. Regarding fatigue, the increase in fatigue observed in the single-color paradigm relative to the dual-modal paradigm was not statistically significant [*b* = +0.90, 95% CI (+0.432, +1.419), *p* = 0.042]. In contrast, the single-motion paradigm yielded a significantly greater increase in fatigue compared to the dual-modal paradigm [*b* = +2.60, 95% CI (+0.824, +4.442), *p* = 0.031]. An ANOVA confirmed the significant effect of paradigm on fatigue, *F*_(2, 18)_ = 8.45, η^2^ = 0.48. *Post-hoc* comparisons using the Tukey HSD test indicated that the dual-modal paradigm resulted in significantly lower fatigue compared to both the single-motion and single-color paradigms.

**Table 2:** Mixed-effects models and analysis of variance results: cognitive load and fatigue across different paradigms.

**Variable**	**Paradigm**	**Mixed-effects**		**ANOVA**	
		**B (95% CI)**	***p*-value**	***F* (df1, df2)**	**η^2^**
Cognitive load	Bimodal	Reference	Reference	15.67 (2, 18)	0.64
	Single color	+2.30 (+0.514, +3.161)	*p* = 0.029		
	Single motion	+13.30 (+10.308, +16.354)	*p* = 0.016		
Fatigue	Bimodal	Reference	Reference	8.45 (2, 18)	0.48
	Single color	+0.90 (+0.432, +1.419)	*p* = 0.042		
	Single motion	+2.60 (+0.824, +4.442)	*p* = 0.031		

Despite the lack of obvious differences in the mean values, the results indicate that the bimodal paradigm effectively alleviates fatigue through the addition of color modalities, showing an advantage in reducing fatigue compared to the single-color and single-motion paradigms, thus validating the design strategy in Section 2.3.

### 3.5 Evaluation of classification performance across different paradigms

We employed the deep learning algorithm EEGNet to calculate classification accuracy for 2-s segments of data under different experimental parameters.

The above study has shown that medium brightness (*M*) and an area ratio of *C* = 0.6 significantly enhance SSVEP responses. Therefore, this study specifically selected these conditions for detailed analysis. Analyzing classification accuracy across different frequencies and data segment durations helps us understand which paradigm performs best under varying experimental conditions, thereby providing a basis for further optimization and design of BCI systems.

**Figure 8:**
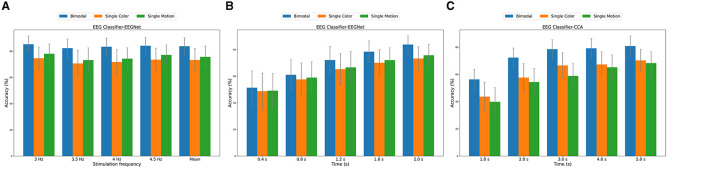
**(A)** EEGNet classification accuracy across different frequencies. **(B)** EEGNet classification accuracy across different data segment durations (0–2 s). **(C)** CCA classification accuracy across different data segment durations (0–5 s).

Figure 8A presents the averaged results across all subjects and all trials, showing the classification accuracy for different frequencies. The bimodal paradigm consistently demonstrated the highest classification accuracy across all frequencies, with the peak accuracy observed at 4.5 Hz (84.18% ± 6.26%). In comparison, the single-color and single-motion paradigms exhibited lower accuracies. The mean accuracy across all frequencies was highest for the bimodal paradigm (83.81% ± 6.52%), followed by single-motion (75.72% ± 8.34%) and single-color (73.31% ± 8.88%) paradigms.

Figure 8B highlights the classification accuracy for different data segment durations, averaged across all subjects, trials, and frequencies. The bimodal paradigm outperformed the other paradigms across all durations. Accuracy increased with longer data segments, peaking at 2 s (83.81% ± 6.52%). The single-motion paradigm showed a similar trend, achieving its highest accuracy (75.72% ± 8.34%) at 2 s, while the single-color paradigm had the lowest peak accuracy (73.31% ± 8.88%) at the same duration.

To further validate the effectiveness of the EEGNet model in SSVEP identification, we introduced the traditional Canonical Correlation Analysis (CCA) method as a comparison. It is important to note that in this study, the CCA method utilized a data segment length of 5 s, as illustrated in Figure 8C, whereas the EEGNet model achieved effective classification with only 2 s of data. However, to ensure a fair comparison, we conducted a classification accuracy analysis of CCA and EEGNet under the same 2-s data segment length. In this 2-s data segment and across different paradigms, the EEGNet method demonstrated higher classification accuracy in all paradigms, particularly in the bimodal paradigm, where its accuracy surpassed that of the CCA method (Bimodal EEGNet: 83.81% ± 6.52%, Bimodal CCA: 72.34% ± 7.08%). The ability of EEGNet to achieve high accuracy with shorter data lengths is primarily attributed to its deep learning architecture, which effectively extracts and leverages complex nonlinear features within the EEG signals. This capability is especially crucial for real-time BCI systems, as real-time applications require the system to make accurate judgments within brief timeframes.

Furthermore, both the traditional CCA classifier and the deep learning EEGNet model confirmed that our bimodal paradigm outperforms the single-color and single-motion paradigms in enhancing SSVEP recognition accuracy. This further supports the importance of the bimodal paradigm in designing efficient and accurate BCI systems.

## 4 Discussion

This study introduced a novel SSMVEP paradigm incorporating bimodal motion-color stimuli, specifically designed for AR glasses. The primary aim was to enhance signal intensity and reduce visual fatigue commonly associated with traditional SSVEP paradigms using light flicker stimuli. Our results demonstrated that the bimodal motion-color paradigm significantly improves the classification accuracy and signal quality of SSMVEP responses, presenting a highly effective solution for BCI applications.

The bimodal paradigm consistently achieved higher classification accuracy compared to single-motion and single-color paradigms across different frequencies and data segment durations. Notably, under medium brightness (M) and an area ratio of *C* = 0.6, the bimodal paradigm achieved the highest accuracy of 83.81% ± 6.52%, significantly outperforming the other paradigms (*p* < 0.001). These findings highlight the paradigm's potential to enhance SSVEP responses and improve BCI performance, offering new avenues for developing more effective BCI systems.

The significant improvement in SNR and classification accuracy with the bimodal paradigm underscores its robustness in inducing SSVEP responses. This paradigm effectively leverages the human visual system's sensitivity to both motion and color changes, engaging the M- and P-pathways in the brain. The higher SNRs and more pronounced evoked responses observed in the frequency domain analyses affirm the paradigm's efficacy in enhancing neural activation. These results suggest that the bimodal paradigm could substantially advance the field of BCI by providing more reliable and less fatiguing user experiences.

However, this study does have several limitations. First, the variability observed in the EEGNet model, as shown in Figures 6, 8, may stem from the model's sensitivity to data quality and quantity. The relatively small sample size of 10 participants, while sufficient for preliminary validation, and the inherent variability of EEG data likely contributed to fluctuations in classification performance. To address this, future research will focus on generating additional EEG datasets and optimizing the EEGNet model to enhance classification accuracy and stability. Moreover, this study focused on specific brightness levels and area ratios, limiting the scope of the parameters explored. Further investigation is needed to explore a wider range of these parameters. Additionally, the use of AR glasses for stimulus presentation may not fully capture other potential application environments. Exploring the effectiveness of the bimodal paradigm across different presentation media, such as virtual reality (VR) and various screen technologies, will be crucial for broader applicability. A notable limitation of this study is the lack of self-report measures for fatigue and cognitive load. The absence of these measures means we could not fully assess the participants' subjective experiences during the experiment. While EEG data provided valuable objective insights, incorporating self-report data in future studies would help us gain a more comprehensive understanding of how participants perceive the stimuli and how fatigue or cognitive load might affect SSVEP responses. We recognize that this limitation may impact the interpretation of our findings and plan to address it in future studies by including self-reported data.

By addressing these limitations, we aim to reduce observed variability and enhance the robustness of EEGNet across diverse experimental conditions. This will help ensure the generalizability and practical applicability of our findings in real-world BCI applications.

## Data Availability

The data analyzed in this study is subject to the following licenses/restrictions. The dataset is not publicly available due to proprietary research and confidentiality agreements. Access to the dataset is restricted and may only be granted upon reasonable request and with permission from the corresponding author or institution. Requests to access these datasets should be directed to Junjie Liu, 1742655433@qq.com.
